# Targeting ubiquitin-specific peptidase 22 in solid tumours: from ubiquitination to immunotherapy

**DOI:** 10.3389/fcell.2026.1792632

**Published:** 2026-03-13

**Authors:** Zhe Li, Liming Wang, Jingyi Liu, Yuanxin Tang

**Affiliations:** 1 Department of Anesthesiology, The Fourth Affiliated Hospital of China Medical University, Shenyang, China; 2 Department of General Surgery, The Fourth Affiliated Hospital of China Medical University, Shenyang, China; 3 Department of Obstetrics and Gynecology, Shengjing Hospital of China Medical University, Shenyang, China

**Keywords:** clinical treatment, ICBs, immune response, metabolism, USP22

## Abstract

As a deubiquitinating enzyme, the function of ubiquitin-specific peptidase 22 (USP22) in tumours has attracted increasing attention. However, the regulatory mechanism underlying USP22’s tumour biological processes has not yet been thoroughly elucidated. Moreover, considering the importance of USP22 in immune regulation and clinical treatment, targeting USP22 to prevent tumour biological processes and improve immunotherapy is worthy of attention. Given the significance of USP22 in immune cell function and tumour progression, clarifying the molecular regulatory network of USP22 in different types of tumours is necessary. In this review, we summarise how USP22 regulates tumour biology and analyse the key role of USP22 in immune regulation to provide new insights into novel therapeutic strategies.

## 1 Overview of USP22

Protein ubiquitination is an important posttranslational modification process in cells and is responsible mainly for regulating multiple biological processes, such as protein activation/inactivation, DNA repair, gene regulation, and signal transduction (Ding et al., 2025; Jiang et al., 2025; Xie et al., 2025). Substrate proteins mediate a range of biological effects through E1-E2-E3 ligase cascades (Henneberg and Schulman, 2021). There are two main types of ubiquitination, monoubiquitination and polyubiquitination. Monoubiquitination is associated mainly with endocytosis, DNA damage, and subcellular protein localisation. Polyubiquitination is mainly responsible for the regulation of protein degradation processes (Pinto et al., 2021). Deubiquitinases (DUBs) are key molecules that regulate ubiquitination. Ubiquitin-specific proteases (USPs) constitute the largest and most diverse group of DUBs, accounting for approximately 60% of DUBs, with their family members being highly conserved (Snyder and Silva, 2021).

**FIGURE 1:**
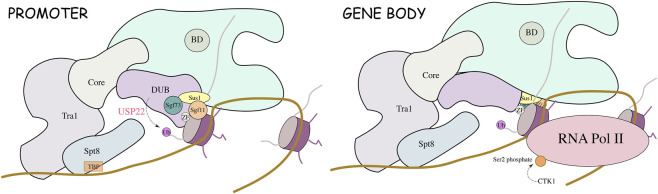
Mechanisms by which the SAGA complex regulates histone ubiquitination.

Ubiquitin-specific peptidase 22 (USP22) belongs to a family of deubiquitinating enzymes and is highly conserved. In yeast, USP22 (called Ubp8) forms the deubiquitinating enzyme module of the Spt-Ada-Gcn5 acetyltransferase complex (SAGA) mainly by binding to Sgf73, Sgf11, and Sus1 (Daniel and Grant, 2007). The SAGA complex regulates various cellular activities through its deubiquitination activity (Daniel and Grant, 2007) (Figure 1). USP22/Ubp8 is composed of two distinct domains, the N-terminal zinc finger domain (ZnF) and the C-terminal catalytic domain, which are responsible for binding Sus1, Sgf11, and Sgf73. The catalytic domain is catalytically active even in the absence of ubiquitin, and Sgf11 and Sgf73 activate Ubp8 catalytic activity and bind to nucleosomes (Bonnet et al., 2008; Kohl et al., 2010; Samara et al., 2010). Thus, it is involved in the regulation of gene transcription and DNA damage repair. These findings suggest that it is critical for maintaining chromatin stability and gene transcription and that its tumour-promoting effect may depend on the regulation of protein stability and chromatin homeostasis.

## 2 The function of USP22 from a tumour microenvironmental perspective

**FIGURE 2:**
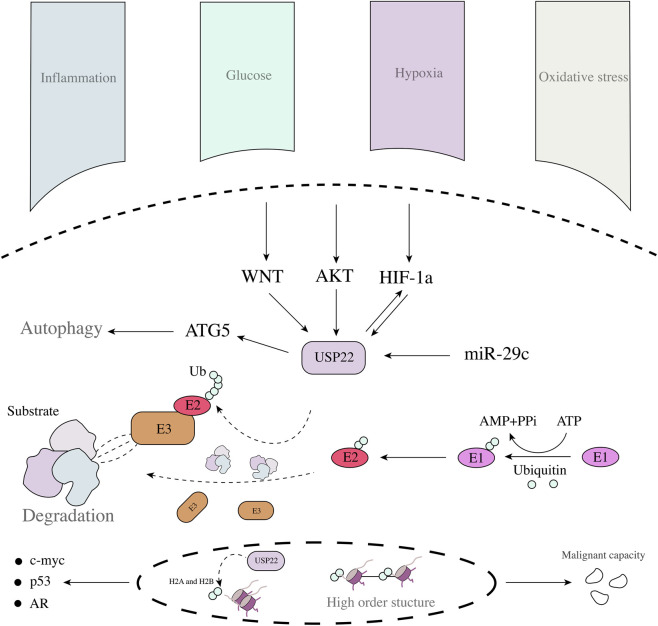
USP22 regulatory network in tumour cells and the role of the ubiquitin-proteasome site.

Solid tumours have unique pathological features. Biological tissue and tumour tissue growth are caused by a lack of blood supply; deep tissue is often unable to meet its sufficient oxygen supply, resulting in the presence of large amounts of oxygen in tumour tissues (Jing et al., 2019; Wicks and Semenza, 2022; Yuen and Wong, 2020). Moreover, tumours are often accompanied by many necrotic areas, leading to persistent inflammation and oxidation product accumulation (Jain et al., 2013; Korbecki et al., 2020; Yamamoto et al., 2023). Owing to their abnormal metabolic characteristics, many metabolic intermediates accumulate in the tumour microenvironment. These features result in tumours with distinct survival pathways. Notably, an abnormal pathological environment can effectively promote expression of USP22, thereby participating in the occurrence and development of tumours (Figure 2). In glioma and hepatocellular carcinoma tissues, hypoxia can promote USP22 expression and trigger tumour progression, which is largely dependent on the involvement of HIF (De Luca et al., 2022; Ling et al., 2020; Qiu et al., 2020). Considering their ability to regulate transcription, HIF and USP22 may be related to the regulation of the relationship between apparent modification and transcriptional activation. An abnormally high-glucose environment also induces the upregulation of USP22 expression (Elumalai et al., 2020; Shi et al., 2016). Notably, even under oxidative stress, knockdown of USP22 expression can inhibit tumour growth (Han et al., 2022). USP22 is important in different stressful environments, indicating that it plays a key role in the carcinogenesis of the tumour microenvironment and that its function may be similar in different environments, especially in solid tumours. The function of USP22 is inseparable in the readjustment of various carcinogenic factors (Table 1). The USP22-related SAGA complex is involved in the regulation of many tumour-related genes including the androgen receptor (AR), the oncogene c-myc, and tumour suppressor p53 during cancer development. USP22 controls the transcription process, triggers the transcription of a variety of downstream genes, regulates the expression of oncogenic proteins, and triggers tumour progression (Feng et al., 2021; Lin et al., 2012). C-myc is a target of USP22, USP22 regulates the malignant ability of cells through c-myc in breast cancer, gastric cancer, and prostate cancer (Kim et al., 2017; Liu et al., 2019; Schrecengost et al., 2014). This finding elucidates the intrinsic regulatory relationship between USP22 and tumour oncogene activation and that this regulatory mode depends on direct regulation and indirect regulation. In addition to its influence on oncogene activation, USP22 can also trigger the activation of multiple signalling pathways, such as AKT and WNT signalling (Zhang et al., 2018), the latter of which may involve USP22 in tumour stemness regulation as well as the key to drug resistance.

**TABLE 1:** Malignant regulatory mechanisms of USP22 in different tumours and experimental design parameters.

Cancer types	Mechanism	*In vivo*/*In vitro*	Cell lines	Functions	Ref
Prostate cancer	XPC	*In vivo* and *In vitro*	LNCaP and C4-2	Cell proliferation and DNA repair	McCann et al. (2020)
Prostate cancer	CCND1	*In vivo* and *In vitro*	PC3	G1/S Cell Cycle Transition	Gennaro et al. (2018)
Lung adenocarcinoma	EGFR/signal kinase	*In vivo* and *In vitro*	H1975 and PC9	Stemness	Zhang et al. (2018)
Lung adenocarcinoma	——	*In vitro*	H1975	Immunosuppression	Han et al. (2020)
Breast cancer	CCND1	*In vivo* and *In vitro*	MCF7	G1/S Cell Cycle Transition	Gennaro et al. (2018)
Breast cancer	c-Myc	*In vitro*	MCF7 and T47D	Tumorigenic activity	Kim et al. (2017)
Breast cancer	ERα	*In vivo* and *In vitro*	MCF7 and MDA-MD-231	Tumour growth and cell proliferation	Wang et al. (2020a)
Hepatocellular carcinoma	E2F6/AKT	*In vivo* and *In vitro*	PLC/PRF5, HEK293T	Tumour growth	Jing et al. (2021)
Hepatocellular carcinoma	COX-2	*In vitro*	HepG2 and Hep3B	Cell proliferation	Xiong et al. (2017)
Hepatocellular carcinoma	SPI1/PD-L1	*In vivo* and *In vitro*	Hep3B and Huh7	Immune evasion	Li et al. (2022)
Hepatocellular carcinoma	PPARγ/ACC/ACLY	*In vivo* and *In vitro*	MHCC97H and MHCC97L	Lipid accumulation and tumorigenesis	Ning et al. (2022)
Non-small cell lung cancer	BMI1	——	Clinical samples	——	Hu et al. (2012)
Non-small cell lung cancer	CCND1	*In vivo* and *In vitro*	H1299	G1/S Cell Cycle Transition	Gennaro et al. (2018)
Non-small cell lung cancer	MDMX/p53	*In vivo* and *In vitro*	A549 and NCI-H460	Tumorigenesis	Ding et al. (2014)
Colon cancer	H2Bub1/p53	*In vivo* and *In vitro*	HCT116	Apoptosis and angiogenesis	Zhang et al. (2023a)
Colon cancer	CCND1	*In vivo* and *In vitro*	HCT116	G1/S Cell Cycle Transition	Gennaro et al. (2018)
Colorectal cancer	SNHG16/miR-132-3p/USP22	*In vivo* and *In vitro*	SW480 and SW620	Proliferation and apoptosis	He et al. (2020)
Colorectal cancer	AP4	*In vivo* and *In vitro*	SW480, SW620, Caco-2, HCT-15, HT29, and LS174T	Metastasis	Jackstadt et al. (2013)
Colorectal cancer	BMI1/INK4a/ARF and AKT	*In vitro*	HCT116	G1 Phase Cell-Cycle Arrest	Liu et al. (2012)
Colorectal cancer	Wnt/β-Catenin	*In vitro*	Caco2, HT29, HCT15, HCT116, SW620 and SW480	Chemoresistance and stemness	Jiang et al. (2018)
Glioblastoma	GSK3β/USP22/KDM1A	*In vivo* and *In vitro*	GSC11, GSC20, GSC23, and GSC262	Stemness	Zhou et al. (2016)
Gastric cancer	c-Myc/NAMPT/SIRT1/FOXO1 and YAP signalling	*In vivo* and *In vitro*	BGC-823 and HGC-27	Growth and metastasis	Liu et al. (2019)
Pancreatic ductal adenocarcinoma	DYRK1A	*In vivo* and *In vitro*	BxPC3 and CAPAN1	Cell proliferation and tumour growth	Bai et al. (2020)
Lung cancer	H2Bub1/p53	*In vivo* and *In vitro*	A549 and H1299	Apoptosis and angiogenesis	Zhang et al. (2023a)
Inflammation-Associated Colorectal Cancer	SPARC (H3K27ac and H2Bub1 Occupancy)	*In vivo* and *In vitro*	HCT116	Local and Systemic Inflammation	Kosinsky et al. (2021)

## 3 Correlation between USP22 and cellular biological function

### 3.1 USP22 and DNA repair

**FIGURE 3:**
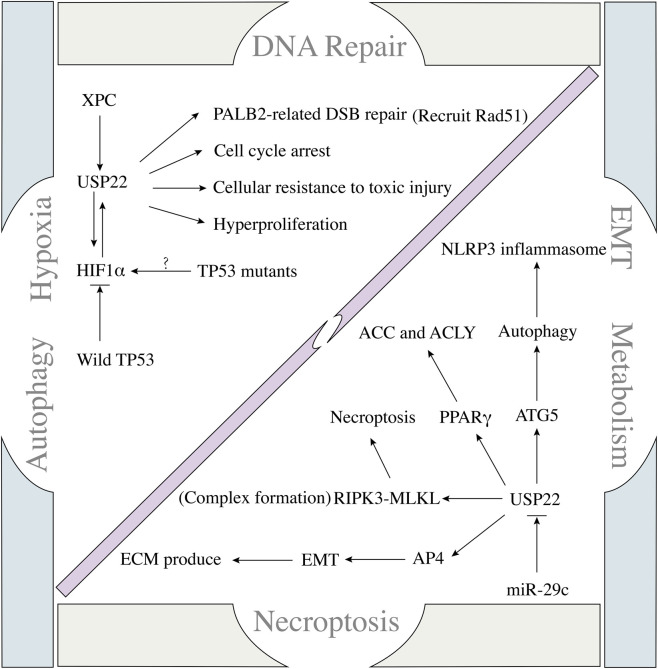
Regulatory network between USP22 and tumour biological processes.

USP22, an oncogene, has been shown to promote tumour progression in a variety of *in vitro* and *in vivo* tumour models, and knockdown of USP22 expression has also been reported to cause G1 blockade to reduce malignant cell proliferation (Lin et al., 2012; Gennaro et al., 2018; Zhang et al., 2017; Zhang et al., 2008). A recent study revealed that USP22 is associated with pancreatic cancer progression and poor prognosis; through transcriptome and ubiquitylome analyses, USP22 is closely related to the cell cycle and DNA repair process, and altering DNA repair nucleotide excision repair (NER) factor-xeroderma pigmentosum group C (XPC) increases the ability of cells to resist toxic injury (McCann et al., 2020). This study further confirmed the oncogenic nature of USP22 and revealed a novel function of USP22 in promoting tumour progression. In addition, USP22 plays an independent role in promoting proliferation in normal tissues; USP22 can trigger the hyperproliferative state of normal mouse prostate cells (McCann et al., 2020). Despite USP22 expression in the mouse prostate, the ability of USP22 to promote proliferation is not obvious; however, independent of its ability to promote proliferation, USP22 also plays an important role in tumorigenesis and development. These findings suggest that USP22 has an independent protumour function and that blocking USP22 expression can inhibit cellular DNA repair and trigger cell cycle arrest to suppress tumorigenesis and progression (Figure 3).

Cells mainly repair DNA double-strand breaks (DSBs) through homologous recombination (HR) repair mechanisms to maintain genome integrity, and the loss of DSB repair function can lead to chromosome loss, carcinogenesis, and apoptosis. Studies have shown that PALB2 is an important protein that maintains HR in lung cancer cells and that USP22 directly interacts with PALB2 through the C-terminal WD40 domain to trigger the catalytic activity of USP22 and recruit Rad51 to participate in the process of DSB repair (Nardi et al., 2020). These findings provide new insight into chemoresistance in lung cancer.

The role of USP22 in tumour drug resistance has been clarified by another study of lung cancer. Chemoresistance in patients with advanced non-small cell lung cancer (NSCLC) is a key factor in clinical treatment failure (Fennell et al., 2016). However, the underlying mechanisms are complex. In a study on lung cancer, the expression of USP22 and the downstream proteins γH2AX and SIRT1 was upregulated in cisplatin-resistant A549/CDDP cells. USP22 can induce cisplatin resistance by triggering histone H2A deubiquitination, exposing histone H2AX^Ser139^ phosphorylation sites, and enhancing the DNA repair capacity of USP22 (Wang et al., 2017). Conversely, USP22 can decrease the acetylation of Ku70 by inducing SIRT1 deubiquitination to promote its expression, leading to inhibition of the BAX-mediated apoptosis process and cisplatin resistance (Wang et al., 2017). This study reveals the dual mechanism of USP22, which is crucial for understanding the drug resistance process in lung cancer patients.

### 3.2 USP22 and the hypoxic response

Hypoxia is a characteristic carcinogenic factor in solid tumours, and this process has a complex relationship with USP22. Hypoxia can trigger changes in USP22 and TP53 expression via HIF. In TP53 wild-type hepatocellular carcinoma (HCC) cells, TP53 can block HIF1α-induced upregulation of USP22 expression (Ling et al., 2020). Once TP53 mutations occur, a positive feedback loop between USP22 and HIF1α promotes the stemness of HCC cells (Ling et al., 2020). TP53 mutation, high USP22 expression, and high HIF1α expression were also associated with poor prognosis in HCC patients. Targeting USP22 can effectively increase the sensitivity of tumour-bearing mice to sorafenib (Ling et al., 2020). USP22 has a similar oncogenic effect on glioma cells. Under hypoxic conditions, USP22 expression knockdown effectively blocked the hypoxia-related cancer-promoting process, which was largely achieved through BMI1 (Qiu et al., 2020). Hypoxia can induce USP22 expression in stem cell-derived exosomes and thus deliver it to target cells, which in turn stabilises HIF1α in target cells (Qian et al., 2024). These studies reveal that USP22 is not only a downstream regulator of hypoxia but also an upstream regulator of HIF1α. These findings indicate a direct positive feedback regulation between USP22 and HIF1α, which provides a new possibility for the treatment of solid tumours, especially oxygen-sensitive tumours.

### 3.3 USP22 and autophagy

In pancreatic cancer (PC), miR-29c directly binds to USP22 mRNA, inhibiting its expression and preventing the activation of USP22-related autophagy (Huang et al., 2018). Although USP22 can affect autophagy activation in tumours, the specific mechanism remains unclear. A recent study revealed that knockdown of USP22 expression can effectively induce the activation of the NLRP3 inflammasome. USP22 can interact with the NLRP3 LRR domain and ATG5 (maintaining the overexpression of ATG5), leading to decreased stability and autophagy activation (lysosomal degradation) (Di et al., 2023). Tumour tissues are in a state of continuous inflammation, which is a key factor in tumour proliferation, invasion, metastasis, and treatment resistance. The NLRP3 inflammasome is important for maintaining host immune homeostasis under conditions of stress. However, in some pathological states, excessive activation of the NLRP3 inflammasome can also lead to metabolic disorders and ageing-related diseases, such as gout, atherosclerosis, type 2 diabetes, and Alzheimer’s disease (Davis et al., 2011; Wen et al., 2012). These studies, to some extent, clarify the potential link between USP22 and autophagy and reveal that the ability of USP22 to regulate autophagy is dependent mainly on its direct binding, providing a novel target for clinical treatment.

### 3.4 Reshaping lipid metabolism by USP22

The liver is pivotal for metabolism, and various liver diseases, especially liver tumours, are associated with metabolic abnormalities. HCC is the main type of liver cancer, and a recent study revealed that an imbalance in cellular lipid metabolism is key to HCC progression. Abnormal lipid metabolism is strongly associated with USP22 expression in HCC tissues, and overexpression of USP22 in HCC (MHCC97H) cells can induce lipid accumulation and promote tumorigenesis through interactions with PPARγ (USP22-PPARγ linking helps to maintain USP22 stability by inhibiting Lys48-linked polyubiquitination) (Ning et al., 2022). PPARγ is a key transcription factor that is closely related to the metabolic enzymes ACC and ACLY (Currie et al., 2013; Bartolacci et al., 2022; Yoon et al., 2021). USP22 can drive *de novo* fatty acid synthesis through upregulation of PPARγ-related ACC and ACLY expression (Ning et al., 2022). Metabolic-associated fatty liver disease (MAFLD) has recently been identified as an important cause of HCC progression and tumorigenesis (Anstee et al., 2019; Baffy et al., 2012). HCC is clearly characterised by genetic alterations, including those in the expression of TP53 and CTNNB1, but the underlying molecular mechanisms have not been clarified. *De novo* lipogenesis hyperactivation during the initiation of MAFLD-related HCC tumorigenesis is strongly associated with various biological processes, such as energy production and cell membrane homeostasis (Donnelly et al., 2005; Rohrig and Schulze, 2016). Fatty acid synthesis requires the participation of several key enzymes, including ATP citrate lyase (ACLY), acetyl-CoA carboxylase (ACC), and fatty acid synthase (FASN) (De Martino et al., 2023; Jin et al., 2021; Wedan et al., 2024). These enzymes are responsible for the conversion of citrate to acetyl-CoA, malonyl-CoA, and palmitic acid. Saturated fatty acids are converted to monounsaturated fatty acids after elongation by stearoyl-CoA desaturase (SCD), followed by triglyceride (TG) production (Currie et al., 2013; Rohrig and Schulze, 2016). In HCC patients, ACLY, ACC, and FASN are abnormally highly expressed and are associated with poor prognosis. Intervention in the expression of these proteins by siRNA and small-molecule drugs effectively inhibits cell invasion both *in vitro* and *in vivo* (De Matteis et al., 2018; Pope et al., 2019). However, to date, progress in clinical therapies targeting lipid synthesis has been limited owing to toxicity or complications (Vander, 2011). Therefore, it is important to identify effective targets for fatty acid synthesis. USP22 is the key factor in the regulation of enzymes involved in fatty acid synthesis and may be a novel target for preventing tumour progression through the inhibition of fatty acid synthesis.

### 3.5 USP22 and necroptosis

Necroptosis is a form of programmed cell necrosis that is important for cells to resist external stimuli to maintain homeostasis. In recent years, it has been found to be closely associated with tumour progression. Moreover, an imbalance in necroptosis is also a key factor in many inflammatory diseases. Recent studies have revealed its importance in tumorigenesis and metastasis, suggesting that targeting necroptosis is a possible new method of treatment (Gao et al., 2022; Yan et al., 2022). Over the past few years, ubiquitination has emerged as a crucial factor in the regulation of necroptosis in cancer cells (Lee et al., 2019; Onizawa et al., 2015). The biological mechanism of USP22 has been well characterised owing to its function in monoubiquitination, by which it can control H2A and H2B monoubiquitination and regulate gene transcription (Zhang et al., 2008; Zhao et al., 2008). Typically, TNFα/TNFR1 is a critical activator of RIPK1 phosphorylation and RIPK3 activation, which can induce necroptosis by promoting RIPK3-MLKL complex-related necrosome formation, and USP22 is responsible for RIPK3 K518 ubiquitination to control RIPK3-MLKL complex formation (Roedig et al., 2021; Y et al., 2019; Tong et al., 2022). In the absence of USP22 in HT-29 and HeLa cells, phosphorylation levels of RIPK3 and ubiquitination at K518 increase, resulting in direct and/or indirect regulation of the ubiquitination process by USP22, for example, by regulating the autophosphorylation of RIPK3 or the activity of RIPK3-associated E3 ligases or kinases (Roedig et al., 2021). These findings suggest that USP22 has a similar biological regulatory ability in some tumours and provide a new therapeutic strategy for the development of targeted drugs against USP22. Clarifying the specific mechanisms associated with different aspects of USP22 can provide better references for improving drug efficacy and reducing adverse drug reactions.

### 3.6 USP22 and EMT

Epithelial–mesenchymal transition (EMT) is a dynamic biological process that occurs widely in cellular physiological and pathological states (Bakir et al., 2020; Brabletz et al., 2021; Pastushenko et al., 2018). EMT is considered the key to tumour cell invasion and metastasis, especially in epithelial tumours. The occurrence of EMT is closely related to the occurrence of high-grade tumours, lymph node metastasis, drug resistance and recurrence (Brabletz et al., 2018; Dart, 2023; Lengrand et al., 2023). Although the mechanism of EMT is gradually becoming clear with the emergence of single-cell sequencing technology, the current view is that EMT is a dynamic and continuous process. Clarifying the dynamic changes in different molecules during the phenotypic transformation of cells is important (Derynck and Weinberg, 2019; Villanueva, 2023). USP22 is associated with EMT progression. In TNBC patients and cells, cells with high expression of USP22 have increased resistance to drugs, and the overexpression of USP22 promotes the expression of EMT-related molecules; moreover, when USP22 expression is knocked down or silenced, EMT is prevented. Mechanistically, USP22 interacts with c-myc to maintain its stabilisation through deubiquitination (Li et al., 2023). In addition to its important functions in cancer, it has a similar ability to regulate EMT under physiological and pathological conditions. In NRK-52 cells, USP22 overexpression can increase the mesenchymal phenotype and stimulate the extracellular matrix (ECM) to produce fibronectin, collagen I, and collagen IV. A study revealed that USP22 promotion is dependent mainly on its deubiquitinase activity, by which it can deubiquitinate and stabilise snail (an EMT transcription factor) (Zhao et al., 2023). The function of USP22 depends on its ability to regulate protein deubiquitination. USP22 can also bind to promoter regions. In a colorectal cancer (CRC) model, increased USP22 expression promotes CRC cell metastasis to the lungs of nude mice, as evidenced by the binding of USP22 to the promoter region of AP4, leading to the transcription of AP4 (Li et al., 2017). AP4, a mediator of EMT, promotes EMT and tumour progression related to HCC, lung adenocarcinoma, and CRC (Jackstadt et al., 2013; Chen and Chiu, 2015; Hu et al., 2015). The regulatory role of USP22 in EMT is inseparable from transcriptional modification and protein stability regulation. Blocking the EMT regulatory signal of USP22 may provide new insight into the prevention of tumour metastasis.

## 4 Role of USP22 and immune checkpoints

**FIGURE 4:**
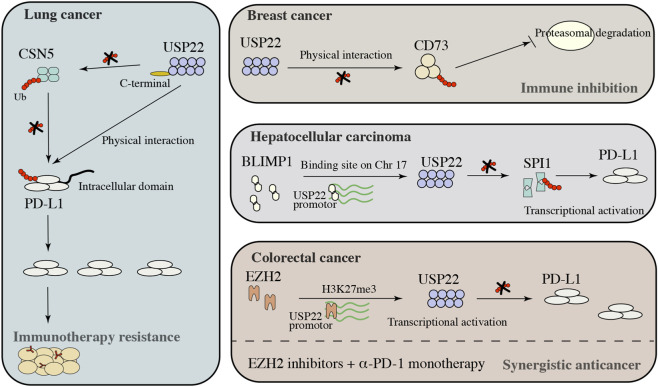
USP22 immune checkpoints in solid tumors.

Significant progress has been made in cancer immunotherapy for melanoma and NSCLC. Programmed death ligand 1 (PD-1) is a 290-amino acid transmembrane glycoprotein belonging to the B7 family. It is an important immune regulatory molecule, and its role in tumour immunotherapy is gradually attracting attention (Hayashi et al., 2024; Wang et al., 2023; Dmello et al., 2023). PD-L1, the ligand of PD-1, is expressed mainly in a variety of immune cells, such as macrophages, activated T cells, B cells, and dendritic cells, especially under inflammatory conditions (Chu et al., 2023; Shapir Itai et al., 2024; Virassamy et al., 2023). Although PD-1/PD-L1 therapy has been gradually applied in clinical practice, it can prevent tumour progression in only some cases. There are clear differences between different tumours; therefore, analysis of the specific mechanism for improving immune therapy is indispensable. This section focuses mainly on the in-depth exploration of different tumours (Figure 4).

### 4.1 Lung cancer

In a study of lung cancer, the regulation of PD-L1 by USP22 depended not only on direct regulation but also on indirect regulation. USP22 can maintain its protein stability by binding to CSN5 (Wang et al., 2020b), and as a major factor responsible for maintaining PD-L1 stabilisation, inhibiting CSN5 expression can sensitise cancer cells to anti-CTLA therapy (Lim et al., 2016). USP22 can directly bind to PD-L1 and inhibit the proteasomal degradation of PD-L1 through deubiquitination (Wang et al., 2020b). Knockdown of USP22 expression can inhibit PD-L1-mediated T cell exhaustion (Wang et al., 2020b), providing a potential molecular basis for improving immune checkpoint blockade therapy. Notably, CSN5 expression can be decreased by curcumin and berberine, leading to an increase in antitumour immune efficacy (Lim et al., 2016; Liu et al., 2020). However, these drugs indirectly regulate PD-L1 expression, and the use of small interfering RNA and small molecule inhibitors decreases USP22 expression, which can be a more efficient and direct control method to restrain PD-L1 expression, thereby increasing the immune treatment effect. The regulatory mechanisms of these bypass pathways may be key to the failure of PD-L1 immunotherapy since cells may have feedback effects in different environments.

### 4.2 Breast cancer

CD73 is a cancer cell-inhibitory immune checkpoint, and its activation inhibits tumour neoantigen-specific immune responses (Jin et al., 2010; Loi et al., 2013; Wang et al., 2008). In a mouse model of breast cancer, the inhibition of CD73 expression decreased tumour growth and metastatic capacity (Gregory et al., 2022). Although a variety of pretranscriptional regulators have been identified, the mechanisms by which CD73 participates in the regulation of CD73 expression at the posttranscriptional level have not been determined. Study revealed that the expression of CD73 was positively correlated with the expression of USP22, however, the loss of USP22 expression did not cause a decrease in CD73 mRNA expression (Gregory et al., 2022). Further analysis revealed that USP22 binds to CD73 and inhibits its ubiquitination interaction, thereby blocking the CD73 proteasomal degradation pathway (Gregory et al., 2022). USP22 protects CD73 from ubiquitin-mediated proteasomal degradation in breast cancer cells, and targeting USP22 can effectively improve immune efficacy.

### 4.3 Colorectal cancer (CRC)

The regulation of PD-L1 expression is a key factor during immunotherapy that largely determines the clinical efficacy of immune checkpoint inhibitors. Recent studies have shown that EZH2 is important for maintaining the stability of the PD-L1 protein in CRC and that USP22 is a key mediator of EZH2 function through an indirect pathway. The inhibition of EZH2 expression increased USP22 transcription, which is the key point to maintaining the stability of the PD-L1 protein and increasing its expression by promoting the stability of PD-L1 (Huang et al., 2024). Notably, administration of an EZH2 inhibitor combined with anti-PD-1 treatment significantly increased the sensitivity of tumours to immunotherapy, and knockdown of USP22 expression enhanced the immunotherapy effect of EZH2 inhibitors in CRC patients (Huang et al., 2024). These findings suggest that a combination of immune checkpoint blockade and EZH2/USP22 inhibitors could result in epigenetic co-optimisation to improve the efficacy and accuracy of cancer therapy.

### 4.4 Hepatocellular carcinoma

During the course of immunotherapy for HCC, most patients do not respond to PD-1/PD-L1 blockade (Sangro et al., 2020; Yang et al., 2021). However, the underlying molecular mechanism remains unclear. Many studies have investigated the PD-L1 control network, which includes a variety of transcription factors, such as HIF-1α, c-MYC, and microRNAs (Song et al., 2020; Wang et al., 2020c). PRDI-BF1 and the RIZ homeodomain (*PRDM*) family play key roles in the pathological stages of different diseases, especially cancer. *PRDM1*, which encodes the BLIMP1 transcription factor, has been implicated in diffuse large B-cell lymphoma progression (Liu et al., 2007; Pasqualucci et al., 2006). BLIMP1 promotes immune evasion in HCC patients by inducing PD-L1 overexpression via the USP22/SPI1 axis, and immunoprecipitation and mass spectrometry (IP/MS) revealed that USP22 interacts with SPI1 mainly through its C-terminal ubiquitin-specific peptidase domain but is not weakly associated with the N-terminal zinc finger domain, moreover, increasing SPI1 expression can promote PD-L1 transcription and result in infiltrating CD8^+^ T cell exhaustion (Li et al., 2022). In the context of HCC immune treatment failure, targeting BMILP1/USP22/SPI1 combined with a PD-L1 monoclonal antibody could improve HCC immunotherapy to provide new strategies and solutions. Single-cell sequencing data analysis revealed that the proportion of the cytotoxic T cell population was significantly decreased in the tissues of patients with low expression of *PRDM1*/BLIMP1, whereas CD8^+^LAG3^+^ exhausted T cells and CD4^+^FOXP3^+^ Tregs were enriched in the tissues of patients with high expression of *PRDM1*/BLIMP1.

## 5 USP22 and immune cell

**FIGURE 5:**
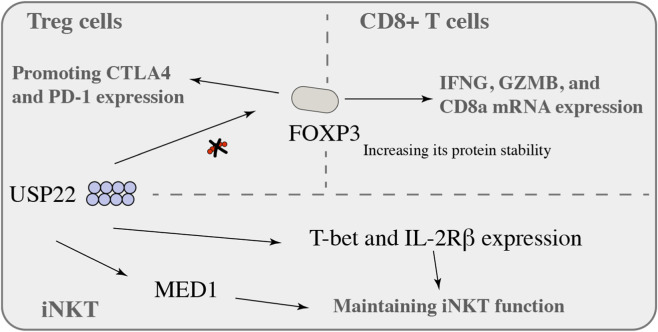
USP22 and immune cells.

In EG7 tumour-bearing mice, USP22 can directly interact with FOXP3, increasing its protein stability, leading to IFNG, GZMB, and CD8a mRNA expression in CD8^+^ T cells increased and strongly enhanced the immune response, USP22 is a regulator of FOXP3 at the transcriptional level and maintains the stability of FOXP3 by regulating FOXP3 deubiquitination and posttranslational modification mechanisms (Cort et al., 2020). USP22-deficient mice have impaired resolution of autoimmune inflammation and enhanced antitumour immune responses *in vivo* (Cort et al., 2020). In addition, in USP22-KO mouse splenic Tregs, the expression of CD25 (FOXP3 targeted gene) was clearly decreased by inhibition of its transcription (Cort et al., 2020). Regulatory T (Treg) cells play a key role in the balance of the immune response. Tregs are generally less stable and are characterized by FOXP3 depletion and a pro-inflammatory phenotype (Zhou et al., 2009). A comprehensive understanding of the regulatory mechanisms that regulate Foxp3 will provide more effective Treg therapeutic strategies (Bailey-Bucktrout and Bluestone, 2011; Overacre-Delgoffe and Vignali, 2018). USP22 is responsible for maintaining FOXP3 stability, which can promote the expression of immunosuppressive Treg markers, including CTLA4 and PD-1, especially in hypoxic microenvironments, and persistent inflammatory activation (Montauti et al., 2022). Interestingly, USP22 and USP21 deletion in Treg cells can play a synergistic role in enhancing antitumour immunity, as determined by a computer-aided drug design (CADD) study in which a USP22-specific small molecular inhibitor, Usp22i-S02, was developed. Usp22i-S02 is an effective small molecular inhibitor that decreases FOXP3 expression in Treg cells in a USP22-dependent manner but has nearly no effect on cell viability, and Usp22i-S02 can improve immunotherapy efficacy and lower immune toxicity in mice (Montauti et al., 2022). USP22 plays important regulatory roles in both tumours and immune cells. Notably, the expression of USP22 in different cell populations may differ, which presents new challenges for the subsequent development of targeted drugs and the use of small-molecule inhibitors (Figure 5).

Invariant natural killer T (iNKT) cells are key nodes of innate and adaptive immunity and are closely related to the efficacy of tumour immunotherapy. iNKT cells express CD1d-specific ligands. Unlike traditional T cells, iNKT cells are stimulated by CD1d presented by CD4^+^CD8^+^ double-positive (DP) thymocytes, together with self-glycolipid antigens (Bendelac et al., 2007). iNKT cells are activated after they quickly grow into NKT1, NKT2, and NKT17 cells and secrete IFN-γ, IL-4-specific T cells, and IL-17-stimulating factor (Engel et al., 2012; Lee et al., 2013; Kadowaki et al., 2001). Recent studies have suggested that the ubiquitin pathway plays a key role in the regulation of iNKT cell development. For example, the ubiquitin-modifying enzyme A20 regulates TCR signalling in T cells through ubiquitination and is also an important regulatory molecule for iNKT maintenance (Drennan et al., 2016). A20 is responsible for the maintenance of NKT cell development and maturation and can regulate the differentiation and survival of NKT1 and NKT2 but not NKT17 subsets through NF-κB (Kojo et al., 2009). These findings suggest that ubiquitination is closely related to the maintenance of immune cell function. Recent studies have revealed that USP22 is a key protein in the maintenance of iNKT function (Zhang et al., 2020). USP22 inhibits the monoubiquitination of H2A but not H2B, leading to T-bet and IL-2Rβ expression in iNKT cells (key molecules in iNKT development). USP22 interacts with another auxiliary transcription activator, MED1, to maintain iNKT cell function (Zhang et al., 2020). In addition, USP22 interacts with c-Myc in tumour cells, and c-Myc is an important regulator of iNKT development (Zhang et al., 2008; Mycko et al., 2009; Dose et al., 2009).

## 6 Crucial role of USP22 in drug resistance

Lenvatinib is a tyrosine kinase inhibitor (TKI), and its pharmacological effect is achieved mainly through the inhibition of VEGFR1-3, FGFR1-4, PDGFR-α, KIT, and RET, thereby preventing tumour progression (Guo et al., 2025; Lu et al., 2024). In the REFLECT study, compared with the first-line drug sorafenib, lenvatinib did not significantly improve overall survival but did increase the progression-free survival rate (Yamashita et al., 2020). Therefore, sorafenib has become the second approved first-line treatment for advanced HCC after sorafenib (Yau et al., 2025). Nevertheless, among HCC patients treated with lenvatinib monotherapy, more than 60% experience progression within 1 year, and the fundamental reason for this is the emergence of drug resistance (Singal et al., 2021). In an HCC model, knockdown of USP22 expression can promote ubiquitin binding to HIF-1α, leading to a decrease in HIF-1α expression, and the stabilisation of HIF-1α expression by USP22 further enhances the survival ability and drug resistance of tumour cells under hypoxic conditions (Shan et al., 2024). Interestingly, USP22 can modulate the ubiquitination of EZH2, thereby leading to the transcriptional silencing of MHC-I gene expression and further causing tumour immune evasion and resistance to checkpoint blockade (Liu et al., 2026). Consequently, USP22 acts as a biomarker for evaluating the efficiency of immunotherapy and a potential therapeutic line.

## 7 Potential substances for modulation of USP22 signalling to improve immune therapy

In the past few years, it has been reported that as a natural substance, rottlerin, can target a variety of tumours and has good antitumour effects. Regarding its mechanism of action, rottlerin can control the function of a variety of enzymes, transcription factors, and signalling molecules in cancer cells (Ma et al., 2018; Maioli et al., 2018). In a structural and biological study, rottlerin (IC50 = 2.53 μM) was found to be a highly selective and potent USP22 inhibitor (Zhang et al., 2023b). Functional experiments revealed that the level of H2B ubiquitination increased after the administration of rottlerin, while the degradation of SIRT1 and PD-L1 increased in a USP22-dependent manner and the polyubiquitination of endogenous PD-L1 and SIRT1 increased (Zhang et al., 2023b). In an *in vivo* syngeneic tumour model, rottlerin exhibited potent antitumour activity, accompanied by enhanced T cell infiltration into tumour tissues. Molecular dynamics (MD) analysis revealed that the Leu475 and Arg419 residues of USP22 are targets of the inhibitor (Zhang et al., 2023b). This study established a potent USP22-specific inhibitor that modulated the expression of immune checkpoint molecules to demonstrate potent immune regulation.

Molecular dynamics studies have screened morusin, another small-molecule inhibitor of USP22. Recently, morusin was shown to have potent antitumour activity, which is partially dependent on multiple pathways, such as MAPK and mTOR signalling (Wu et al., 2023; Zhang et al., 2024; Zhou et al., 2021). Like rottlerin, morusin is a potent USP22 inhibitor. In HCT116 and A375 cells, morusin significantly increased the monoubiquitination of histones H2A and H2B and decreased the protein expression of SIRT1 and PD-L1 (Zhang et al., 2023b). Considering that USP22 is also a regulator of signalling by a variety of kinases (Jing et al., 2021; Jiang et al., 2023), the tumour-suppressive effect of morusin may act synergistically by directly acting on USP22 as well as by blocking downstream signals of USP22.

Because USP22 contains putative catalytic domains (Cys, His, and Asp) that are highly conserved from yeast to humans, on the basis of homology modelling studies, the use of CADD has led to the development of a specific small-molecule inhibitor of USP22 (Usp22i-S02), which can significantly reduce FOXP3 expression in Treg cells without affecting cell viability, thereby significantly improving the efficacy of immunotherapy (Montauti et al., 2022). However, it should be noted that the inhibitory effect of Usp22i-S02 was verified mainly *in vitro*, and whether the inhibitory effect is selective once applied to the whole body should also be considered, although this study confirmed that Usp22i-S02 has only minimal toxicity and exhibits good antitumour immunity in mice.

## 8 Future directions and potential applications

Small-molecule drugs and gene interference agents for USP22 are being developed, but the clinical use of these targeted drugs still requires long-term verification. In addition, with the development of drugs targeting USP22, a variety of downstream regulatory networks can be used as new drug targets. For example, USP22 can activate a variety of signalling pathways, such as mTOR, YAP, and WNT signalling (Liu et al., 2019; Jing et al., 2021; Deng et al., 2023). Blocking downstream signalling cascades may provide new strategies for improving tumour immunotherapy; however, it should be noted that USP22 or its downstream small-molecule inhibitors do not have the ability to target cell subsets, which may lead to an overall effect that is inconsistent with expectations. The development of single-cell transcriptomes and space technology can provide novel insights into this defect.
